# Sleeve gastrectomy *versus* Roux-en-Y-gastric bypass in patients with body mass index over 50 kg/m^2^: international multicentre cohort

**DOI:** 10.1093/bjsopen/zrag028

**Published:** 2026-04-29

**Authors:** Lars Kollmann, Ilan Rosenblum, Adisa Poljo, Pascal Probst, Markus K Muller, Piotr Kalinowski, Muhammed S Dalkılıç, Abdullah Şişik, Johanna Betzler, Mirko Otto, Stephanie Taha-Mehlitz, Beat P Müller, Daniel M Frey, Bassey Enodien, Emanuel Burri, Reinhard Stoll, Otto Kollmar, Robert Rosenberg, Florian Ponholzer, Johan Friso Lock, Sven Flemming, Erik Stenberg, Johan Ottosson, Ellen Andersson, Torsten Olbers, Ralph Peterli, Florian Seyfried, Anas Taha

**Affiliations:** Department of General-, Visceral-, Transplantation-, Vascular-, and Pediatric Surgery, University Hospital Wuerzburg, Wuerzburg, Germany; Department of Visceral Surgery, Cantonal Hospital Baselland, Liestal, Switzerland; Department of Visceral Surgery, Clarunis, University Center for Gastrointestinal and Liver Diseases, St Clara Hospital and University Hospital, Basel, Switzerland; Medical Faculty, Johannes Kepler University Linz, Linz, Austria; Department of Surgery, Cantonal Hospital Thurgau, Frauenfeld, Switzerland; Department of Surgery, Cantonal Hospital Thurgau, Frauenfeld, Switzerland; Department of General, Transplant and Liver Surgery, Medical University of Warsaw, Warszawa, Poland; Department of General Surgery, School of Medicine, Marmara University, Istanbul, Turkey; Health Sciences Faculty, Gedik University, İstanbul, Turkey; Department of Surgery, Medical Faculty Mannheim, Universitätsmedizin Mannheim, Heidelberg University, Mannheim, Germany; Department of Surgery, Medical Faculty Mannheim, Universitätsmedizin Mannheim, Heidelberg University, Mannheim, Germany; Department of Visceral Surgery, Clarunis, University Center for Gastrointestinal and Liver Diseases, St Clara Hospital and University Hospital, Basel, Switzerland; Department of Visceral Surgery, Clarunis, University Center for Gastrointestinal and Liver Diseases, St Clara Hospital and University Hospital, Basel, Switzerland; Department of Visceral Surgery, Cantonal Hospital Baden, Baden, Switzerland; Department of Visceral Surgery, Cantonal Hospital Glarus, Glarus, Switzerland; Department of Gastroenterology and Hepatology, Cantonal Hospital Baselland, Liestal, Switzerland; Department of Visceral Surgery, Cantonal Hospital Baselland, Liestal, Switzerland; Department of Visceral Surgery, Cantonal Hospital Baselland, Liestal, Switzerland; Department of Visceral Surgery, Cantonal Hospital Baselland, Liestal, Switzerland; Department of Visceral, Transplant and Thoracic Surgery, Center of Operative Medicine, Medical University of Innsbruck, Innsbruck, Austria; Department of General-, Visceral-, Transplantation-, Vascular-, and Pediatric Surgery, University Hospital Wuerzburg, Wuerzburg, Germany; Department of General-, Visceral-, Transplantation-, Vascular-, and Pediatric Surgery, University Hospital Wuerzburg, Wuerzburg, Germany; Department of Surgery, Faculty of Medicine and Health, Örebro University, Örebro, Sweden; Department of Surgery, Faculty of Medicine and Health, Örebro University, Örebro, Sweden; Department of Surgery and Department of Clinical and Experimental Medicine, Linköping University, Norrköping, Sweden; Department of Surgery and Department of Clinical and Experimental Medicine, Linköping University, Norrköping, Sweden; Department of Clinical Research, University Hospital Basel, Basel, Switzerland; Department of General-, Visceral-, Transplantation-, Vascular-, and Pediatric Surgery, University Hospital Wuerzburg, Wuerzburg, Germany; Department of Visceral Surgery, Cantonal Hospital Baselland, Liestal, Switzerland; Department of Surgery, Department of Surgery, Brody School of Medicine, East Carolina University, Greenville, North Carolina, USA; Department of Biomedical Engineering, University of Basel, Basel, Switzerland

**Keywords:** weight loss, diabetes remission, obesity class IV

## Abstract

**Background:**

Patients with initial body mass index > 50 kg/m^2^ are vastly under-represented in randomized clinical trials demonstrating similar weight loss and diabetes remission rates after sleeve gastrectomy and Roux-en-Y gastric bypass.

**Methods:**

Propensity score matching 1 : 1 was used to compare outcomes regarding weight loss and diabetes control after sleeve gastrectomy and Roux-en-Y gastric bypass in patients with body mass index > 50 kg/m^2^ between 2012 and 2022 in a cohort from 13 centres in six European countries. The primary endpoint was percentage total bodyweight loss; secondary endpoints were diabetes remission rate and rate of persistent body mass index > 40 kg/m^2^.

**Results:**

In total, 3976 of 8160 patients were matched and included in the analysis (1988 in each group). Median age at baseline was 40.0 (range 16–76) years in the sleeve gastrectomy group and 39.5 (15–71) years in the Roux-en-Y gastric bypass group. Median body mass index at baseline was 56.2 (range 50.0–100.0) and 54.3 (50.0–83.9) kg/m^2^, respectively (*P* < 0.001). The follow-up rate was 70.5% at 1 year and 24.4% at 5 years. Percentage total bodyweight loss at 1 and 5 years after sleeve gastrectomy was 30.2 (2.2–63.7) and 25.4 (–4.8 to 56.0)%, respectively, *versus* 31.2 (7.4–54.5) and 28.2 (−6.6 to 62.9)% in the Roux-en-Y gastric bypass group (*P* < 0.001 between groups in both time points). The prevalence of persistent body mass index > 40 kg/m^2^ after 1 and 5 years was 42.7 and 57.6%, respectively, after sleeve gastrectomy *versus* 24.5 and 39.2% after Roux-en-Y gastric bypass (*P* < 0.001 between groups in both time points). A 5-year follow-up, the prevalence of a pathological haemoglobin A1c level (> 6.5%) was 12.9% after sleeve gastrectomy and 11.6% after Roux-en-Y gastric bypass (*P* = 0.323).

**Conclusion:**

This study suggests that Roux-en-Y gastric bypass results in greater weight loss than sleeve gastrectomy in patients with body mass index > 50 kg/m^2^, whereas improvements in diabetes appear comparable between procedures.

## Teaser

This retrospective multicentre cohort study included 3978 patients after sleeve gastrectomy and Roux-en-Y-gastric bypass (RYGB) with initial body mass index > 50 kg/m^2^. The early and mid-term results (1 and 5 years) showed superiority for the RYGB regarding weight loss and sustainability. Diabetes remission rates did not differ.

## Introduction

A recent report^1^ from the USA suggested a drastic increase in prevalence of people living with obesity, with the steepest increase in those with body mass index (BMI) > 50 kg/m^2^. Among the various bariatric procedures available, laparoscopic sleeve gastrectomy (SG) and Roux-en-Y gastric bypass (RYGB) are currently the most commonly used worldwide^2^.

Long-term data from two randomized clinical trials (RCTs)^3,4^ comparing the efficiency of SG *versus* RYGB showed similar outcomes for both procedures in terms of weight loss over a 10-year follow-up^5,6^. Mid-term results from a third, large RCT^7^ have also indicated high perioperative safety for both procedures. However, the majority of patients included in these studies^5–8^ had BMI < 50 kg/m^2^. Previous RCTs^5,6^ further reported a high rate of conversion surgery, exceeding 30%, in the SG group, primarily owing to secondary gastro-oesophageal reflux disease (GORD) and/or insufficient weight loss.

Suboptimal weight loss and/or weight regain are common after SG^3–6^, but they have also been reported following RYGB, especially in patients with high initial BMI^9^. However, RCTs or real-world data from prospective registry studies comparing outcomes of SG and RYGB in cohorts with an initial BMI > 50 kg/m^2^ remain scarce.

The aim of this study was to compare the efficacy of SG and RYGB for weight loss and diabetes improvement after 1 and 5 years in patients with initial BMI > 50 kg/m^2^, using data from a multicentre European cohort.

## Methods

### Patients

This was a retrospective analysis of prospectively collected data for consecutive patients with primary BMI > 50 kg/m^2^ who underwent RYGB or SG at 13 participating centres from 2012 to 2022. Data from Sweden were based on anonymized data from the Scandinavian Obesity Surgery Registry, a national research and quality registry including data from nearly all patients undergoing metabolic bariatric surgery (MBS) nationwide. This registry is continuously validated with high validity of the included data^10^. Data from two German centres were collected prospectively from the national quality and research registry of the German Society of General and Visceral Surgery. Data from Switzerland were collected prospectively in the local anonymized databases of the participating centres. Data from Austria, Poland, and Turkey were collected retrospectively from the local hospital information systems. The distribution of procedures and participating centres is summarized in *Table S1*.

The manuscript was prepared according to SQUIRE^11^. Informed consent was waived with respect to the use of anonymized data. This study was approved by the Zurich Ethics Committee, Switzerland (BASEC-Nr 2022-00659). All centres approved the study.

### Data extracted

Data extracted from the national registries included baseline characteristics, such as age, sex, BMI, diabetes status (diabetes defined as haemoglobin (Hb) A1c level ≥ 6.5%), insulin dependence (but not dosage or other medication), and type of surgical procedure (SG or RYGB). Follow-up data at 1, 2, and 5 years included BMI, diabetes status, percentage total bodyweight loss (%TBWL), insufficient weight loss (defined as BMI > 40 kg/m^2^ for a cohort with primary BMI > 50 kg/m^2^) and follow-up rates^9^. Because of inconsistent data sets, especially missing values for weight and height, as well as fasting glucose levels, the authors decided to assess only BMI, weight loss, and diabetes status in this analysis.

### Outcomes

The primary endpoint was %TBWL at 1 and 5 years after surgery. Secondary endpoints included remission of type 2 diabetes (T2D), defined by HbA1c level < 6.5%^12^, and persistent BMI > 40 kg/m^2^ at 1 and 5 years following SG and RYGB. %TBWL was analysed in quartiles (< 10, 10–20, 20–30, and > 30%).

### Statistical analysis

Descriptive data are reported as mean(standard deviation), unless otherwise stated. The main analysis comprised a comparison of propensity score-matched groups of patients undergoing SG and RYGB. The propensity variable for 1 : 1 matching with the nearest neighbour was calculated by logistic regression analysis incorporating the selected co-variables. A caliper of < 0.05 of the standard deviation of the logit of the propensity score was accepted. Co-variables for propensity score matching were age, sex, T2D, and BMI as they were the most important factors regarding the outcome variables, and were the most complete data available for the majority of the patients. Validation of the matching criteria was assessed with standardized mean differences and shown as Cohen’s d. Comparisons between the cohorts were performed using the χ^2^ test, Fisher’s exact test, Mann–Whitney *U* test or one-way analysis of variance, in accordance with data scale and distribution. The level of statistical significance was set at 0.05 (2-sided). All statistical analyses were also carried out for the baseline cohort (including patients with missing follow-up) and only for the subgroup of patients with completed 5-year follow-up to control for possible selection bias (*supplementary tables*). Patients with missing baseline data for the matching criteria were excluded from the analysis. All statistical analyses were undertaken using SPSS^®^ version 29 (IBM, Armonk, NY, USA).

## Results

**Fig. 1:**
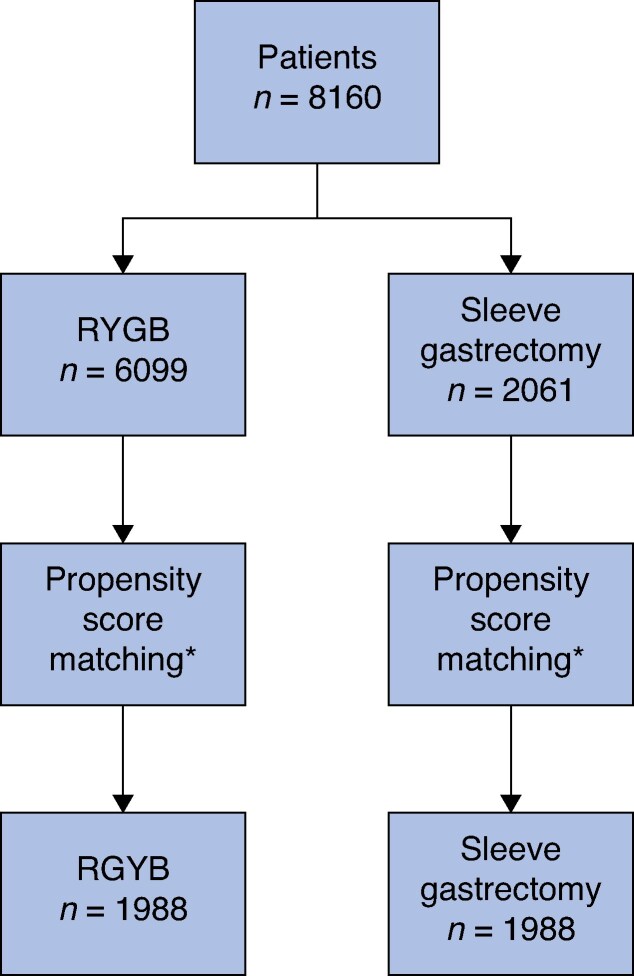
Study flow chart *Co-variables for propensity score matching were age, sex, T2D, and BMI. RYGB, Roux-en-Y gastric bypass.

Overall, data for 8160 patients were retrieved from the database (2061 SG, 6099 RYGB). After propensity score matching, 3976 patients remained as the study cohort (1988 in each groups) (*Fig. 1*). There were no statistically significant and clinically relevant differences at baseline regarding age (median 39.9 years), sex distribution (65.1% women), or prevalence of T2D (26.4%) (*Table 1*). Median baseline BMI was 55.5 (range 50.0–100.0) kg/m^2^ overall, 56.2 (50.0–100.0) kg/m^2^ in the SG group and 54.3 (50.0–83.9) kg/m^2^ in the RYGB group (*P* < 0.001). Follow-up rates at 1 and 5 years were 70.5 and 24.4% overall; they were 71.1 and 69.9% at 1 year (*P* = 0.212), and 21.2 *versus* 27.6% at 5 years (*P* = 0.001), after SG and RYGB, respectively.

**Table 1:** Baseline characteristics

Patient characteristics	Total cohort (*n* = 3976)	Sleeve gastrectomy (*n* = 1988)	RYGB (*n* = 1988)	SMD (Cohen’s d)
Age median (range)	39.9 (15–76)	40.0 (16–76)	39.5 (15–71)	0.063
**Sex**				0.002
Male	1388 (34.9%)	693 (35.0%)	693 (34.9%)	
Female	2588 (65.1%)	1293 (65.0%)	1295 (65.1%)	
BMI at baseline (kg/m^2^), median (range)	55.5 (50.0–100.0)	56.2 (50.0–100.0)	54.3 (50.0–83.9)	0.370
**Diabetes at baseline**	1050 of 3976 (26.4%)	526 of 1988 (26.5%)	524 of 1988 (26.4%)	0.002
Insulin-dependent (1159 patients available)	70 of 1159 (6.0%)	49 of 903 (5.4%)	21 of 256 (8.2%)	

Values are *n* (%) unless otherwise stated. RYGB, Roux-en-Y gastric bypass; SMD, standardized mean difference; BMI, body mass index.

At 1 year after operation, median BMI had decreased from 55.5 (50–100) to 38.3 (22.0–64.7) kg/m^2^ overall, from 56.2 (50–100) to 39.3 (22.0–64.7) kg/m^2^ in the SG group, and from 54.3 (50–83.9) to 37.5 (23.7–59.7) kg/m^2^ in the RYGB group (*P* < 0.001 for SG *versus* RYGB) (*Table 2*). Accordingly, %TBWL was 30.7% overall, 30.2% after SG and 31.2% after RYGB (*P* < 0.001). At 1 year, 943 of 2803 patients (33.6%) overall had BMI > 40 kg/m^2^, including 603 of 1413 (42.7%) in the SG group and 340 of 1390 (24.5%) in the RYGB group (*P* < 0.001). T2D persisted at 1 year in 203 of 2412 patients (8.4%) overall, 82 (8.8%) in the SG group and 121 (8.2%) in the RYGB group (*P* = 0.312).

**Table 2:** Results at 1 year

	Total cohort (*n* = 2803)	Sleeve gastrectomy (*n* = 1413)	RYGB (*n* = 1390)	P*
BMI at 1 year (kg/m^2^), median (range)	38.3 (22.0–64.7)	39.3 (22.0–64.7)	37.5 (23.7–59.7)	< 0.001†
Diabetes at 1 year (2412 patients available)	203 of 2412 (8.4%)	82 of 930 (8.8%)	121 of 1482 (8.2%)	0.312
%TBWL at 1 year, median (range)	30.7 (2.2–63.7)	30.2 (2.2–63.7)	31.2 (7.4–54.5)	< 0.001†
BMI > 40 kg/m^2^ at 1 year	943 of 2803 (33.6%)	603 of 1413 (42.7%)	340 of 1390 (24.5%)	< 0.001
BMI > 50 kg/m^2^ at 1 year	100 of 2803 (3.6%)	80 of 1413 (5.7%)	20 of 1390 (1.4%)	< 0.001
Follow-up rate at 1 year (%)	70.5	71.1	69.9	0.212

Values are *n* (%) unless otherwise stated. RYGB, Roux-en-Y gastric bypass; BMI, body mass index; %TBWL, percentage total bodyweight loss. *χ^2^ test or Fisher’s exact test, except †Mann–Whitney *U* test.

At 5 years, median BMI was 40.0 (20.0–67.2) kg/m^2^ overall, 41.6 (22.0–67.2) kg/m^2^ in the SG group and 38.7 (20.0–63.8) kg/m^2^ in the RYGB group (*P* < 0.001) (*Table 3*). Accordingly, %TBWL was 27.0% overall, 25.4% after SG and 28.2% after RYGB (*P* < 0.001). At 5 years, obesity with BMI > 40 kg/m^2^ persisted in 458 of 970 patients (47.2%) overall, including 243 of 422 (57.6%) in the SG group and 215 of 548 (39.2%) in the RYGB group (*P* < 0.001). The prevalence of T2D was 116 of 849 patients (13.7%) overall, 41 (12.9%) in the SG group and 75 (11.6%) in the RYGB group (*P* = 0.323).

**Table 3:** Results at 5 years

	Total cohort (*n* = 970)	Sleeve gastrectomy (*n* = 422)	RYGB (*n* = 548)	P*
BMI at 5 years (kg/m^2^), median (range)	40.0 (20.0–67.2)	41.6 (22.0–67.2)	38.7 (20.0–63.8)	< 0.001†
Diabetes at 5 years (849 patients)	116 of 849 (13.7%)	41 of 318 (12.9%)	75 of 647 (11.6%)	0.323
%TBWL at 5 years, median (range)	27.0 (−6.6 to 62.9)	25.4 (−4.8 to 56)	28.2 (−6.6 to 62.9)	< 0.001†
BMI > 40 kg/m^2^ at 5 years	458 of 970 (47.2%)	243 of 422 (57.6%)	215 of 548 (39.2%)	< 0.001
BMI > 50 kg/m^2^ at 5 years	75 of 970 (7.7%)	57 of 422 (13.5%)	18 of 548 (3.3%)	< 0.001
Follow-up rate at 5 years (%)	24.4	21.2	27.6	0.001

Values are *n* (%) unless otherwise stated. RYGB, Roux-en-Y gastric bypass; BMI, body mass index; %TBWL, percentage total bodyweight loss. *χ^2^ test or Fishers exact test, except †Mann–Whitney *U* test.

**Fig. 2:**
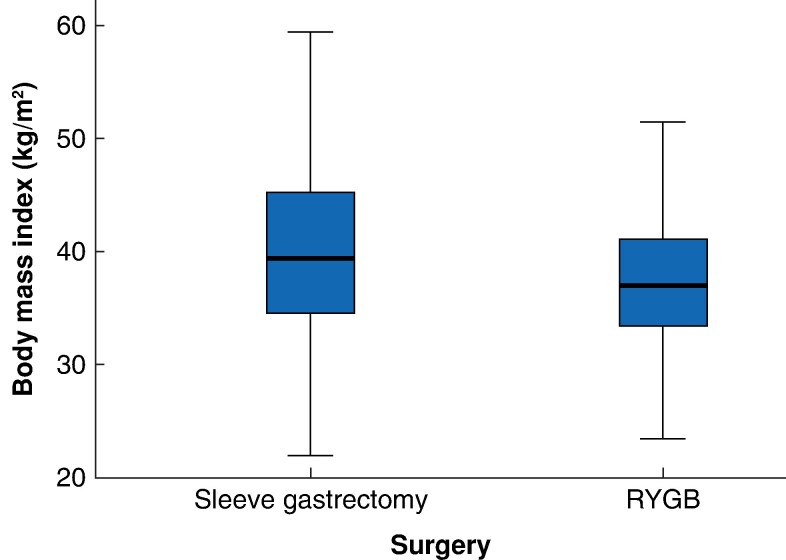
Body mass index distribution 5 years after sleeve gastrectomy and RYGB Median values, interquartile ranges, ranges, and outliers are represented by bold lines, boxes, error bars, and circles, respectively. RYGB, Roux-en-Y gastric bypass.

**Fig. 3:**
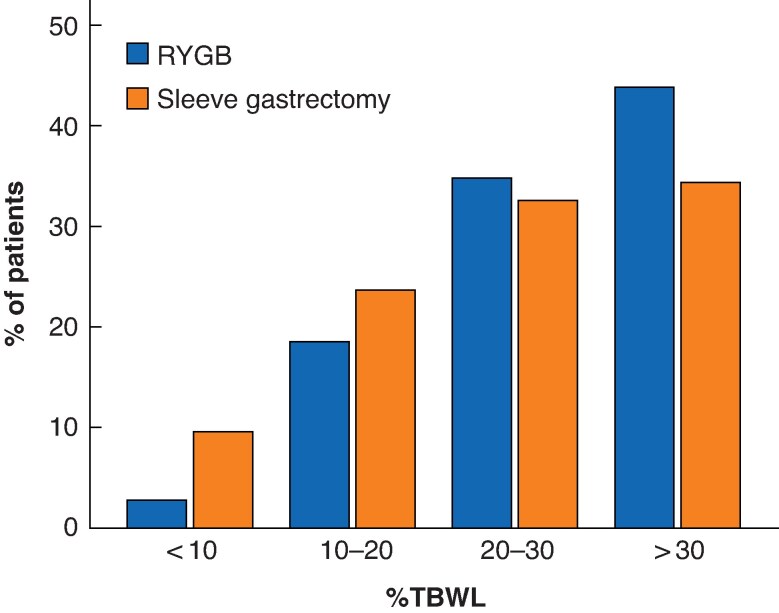
Distribution of %TBWL after sleeve gastrectomy and RYGB %TBWL, percentage total bodyweight loss; RYGB, Roux-en-Y gastric bypass.

BMI distributions for SG and RYGB at 5 years are shown in *Fig. 2*. The proportion of patients achieving TBWL < 10% at 5 years was 2.8% in the RYGB group *versus* 9.5% in the SG group (*P* < 0.001); 18.5 *versus* 23.6%, respectively, achieved 10–20% TBWL; 34.9 *versus* 32.5% achieved 20–30% TBWL; and 43.9 *versus* 34.4% achieved > 30% TBWL (*P* < 0.001) (*Fig. 3*).

### Supplementary analyses

Baseline characteristics and outcomes in the overall cohort of 8160 patients showed trends in %TBWL, BMI, and the rate of patients with suboptimal weight loss similar to those observed in the propensity score-matched cohort (*Tables S2*–*S4*). Results for the subgroup of patients with complete 5-year follow-up did not differ from those for the overall cohort (*Tables S5* and *S6*).

## Discussion

In this large multicentre cohort study examining the effectiveness of SG and RYGB in patients with BMI > 50 kg/m^2^, the initial weight loss at 1 year —31.2% TBWL after RYGB and 30.2% after SG—showed no clinically relevant difference between the groups and is consistent with previous reports^5–7,13^. At 5 years, RYGB resulted in a lower BMI (38.7 *versus* 41.6 kg/m^2^) and correspondingly greater %TBWL (28.2 *versus* 25.4%). This is presumably clinically relevant, especially with respect to the rates of persistent BMI > 40 and > 50 kg/m^2^ at both 1 and 5 years. Notably, the proportion of patients achieving insufficient weight loss was smaller after RYGB than SG. Despite these differences in weight outcomes, the prevalence of T2D remained similar between the two groups.

Although no difference was observed in maximum weight loss, 5-year weight loss was more favourable after RYGB, consistent with previous findings^14,15^. Both groups achieved clinically relevant and sufficient weight loss at both follow-up times, with modest recurrent weight gain after both procedures at 5 years. Similarly, recurrence of T2D increased at 5 years compared with the 12-month follow-up.

These results are in line with the findings from the SM-BOSS and the SLEEVEPASS trials^3–6^, demonstrating the efficiency of both procedures in patients with BMI > 50 kg/m^2^. Other studies investigating weight loss after SG and RYGB in patients with BMI > 50 or > 60 kg/m^2^ have reported similar outcomes. A retrospective study from Arapis *et al*.^16^ found comparable weight loss for patients with BMI > 60 kg/m^2^ after 4 years of follow-up, with no differences between SG and RYGB. A meta-analysis by Wang *et al*.^17^ reported similar results with superior percentage excess weight loss (%EWL) for RYGB at 12 months, but no difference at 24 months. The largest study to date on this topic, by Jain *et al.*^18^ in 2018, included > 3000 patients, with a large subgroup of > 450 patients with BMI > 50 kg/m^2^, and also found no difference in %EWL for SG and RYGB at 24-month follow-up.

The most notable difference between the groups in the present study was greater variance in %TBWL and higher rates of persistent BMI > 40 and > 50 kg/m^2^ at both follow-up times in the SG group, indicating long-term weight loss superiority for RYGB. This suggests that mid-term (5-year) weight loss in patients with very high baseline BMI is substantially lower than in cohorts from prospective RCTs with a median BMI of ∼40 kg/m^23–8^. Substantial weight loss is particularly important in patients with BMI > 50 kg/m^2^, as BMI > 40 kg/m^2^ is associated with reduced life expectancy^19^, and MBS has been shown to effectively mitigate these risks^20,21^. As postoperative weight loss largely determines remission of obesity-related problems, achieving sufficient weight loss target is critical. Several studies^3–7^ have demonstrated that RYGB results in greater remission of metabolic disorders such as hyperlipidaemia and T2D compared with SG^3–7^. Both procedures, however, have been shown to improve health-related quality of life and psychosocial functioning^22^.

These findings highlight the need for improved multimodal therapeutic strategies in this patient population, for example by adding obesity management medications upfront or after surgery^23,24^. At least an effect of around 8–9% additional %TBWL has been demonstrated by administration of semaglutide after non-response to MBS^25^. Furthermore, procedures with higher weight loss potential such as one-anastomosis gastric bypass (OAGB) or single-anastomosis duodenoileal bypass with sleeve should be emphasized as they could be a valuable option in these patients^26^. Furthermore, considering conversion surgery from SG to bypass procedures should be an option^27^. Additionally, physicians and surgeons should emphasize long-term results in preoperative consultations to set realistic expectations, as individual outcomes may deviate from overall trends^28^.

In addition, weight-loss trajectories should be interpreted in the context of long-term adverse events. For RYGB, this includes secondary surgical interventions for complications such as internal hernia, which has reported rates of up to 15% if mesenteric defects are not closed^29,30^. For SG, secondary conversion to RYGB owing to persistent GORD or suboptimal weight loss has been reported in approximately 30–40% of patients in RCTs^3–7^ at 5- and 10-year follow-up.

This study has several limitations. It comprised a retrospective analysis with data collected from multiple centres. The number of included patients per centre differed substantially. This may have introduced selection bias. The 5-year follow-up rate was approximately 25%, which is comparable to that in other studies but represents an extra potential source of selection bias which may have influenced the results. To address this, the authors also undertook statistical analyses of the primary cohort before propensity score matching and of the subgroup of patients with complete 5-year follow-up; these showed similar results regarding weight loss and diabetes remission. The results from these cohorts were largely consistent with those of the propensity score-matched group, supporting the reliability of the observed effects, particularly given the large sample size. Therefore, the findings are likely clinically relevant and generalizable. Baseline BMI was higher in the SG group than in the RYGB group despite very strict propensity score matching. This effect was most likely driven by the large sample sizes; nevertheless, bias may exist here. The consistent differences in BMI during follow-up underline the similar weight loss potential. However, T2D remission was assessed based solely on HbA1c levels. Data on use of insulin and other antidiabetic drugs, quality of life, and other associated co-morbidities were not available. This limitation restricts the assessment of their effects on associated medical problems, which is essential for clinical decision-making and optimization of therapeutic strategy.

Furthermore, information was lacking on conversion surgery after SG owing to initial data selection, which may have led to overestimation of the weight loss effect of SG. Additionally, because of the retrospective nature of the study, certain relevant outcomes could not be assessed, such as the incidence of secondary GORD after SG or internal herniation after RYGB.

This study was based on observational data. Although the results imply superior weight loss results for RYGB, causal conclusions cannot be drawn.

To the authors’ knowledge, this is the largest multicentre study investigating patients with BMI > 50 kg/m^2^ undergoing primary MBS, and it represents an attempt to address which procedure may be preferable and whether SG is sufficient as a stand-alone procedure in the long term. The data indicate that SG achieves short-term weight loss and T2D remission comparable to those of RYGB, but is associated with a higher risk of suboptimal weight loss. These results are in line with other previous cohort studies examining this question^18,26^. Some patients with very high BMI may not be suitable for primary RYGB and may undergo SG as part of a staged surgical strategy or could be treated with other bypass procedures such as OAGB^26^. Importantly, requiring subsequent conversion to a bypass procedure should not be viewed negatively. Adjunctive medical treatments may also improve results in this group.

Therefore, the authors believe that careful follow-up for this patient group is essential, and that planned conversion to a bypass procedure may help address recurrent weight gain after SG. They also advocate for a prospective RCT in this patient cohort. Additionally, a study comparing planned conversion to RYGB *versus* OAGB after SG in patients who are not eligible for primary bypass procedures would provide valuable evidence in the field of obesity medicine.

## Collaborators

M. Bartkowiak, M. Przybysz, M. Grąt (Medical University of Warsaw, Warsaw, Poland), M.-C. Neuschmid, and C. Bogensperger (Medical University of Innsbruck, Innsbruck, Austria)

## Supplementary Material

zrag028_Supplementary_Data

## Data Availability

The datasets used and/or analyzed during the current study are available from the corresponding author on reasonable request.

## References

[1] Kachmar M, Albaugh VL, Yang S, Corpodean F, Heymsfield SB, Katzmarzyk PT et al Disproportionate increase in BMI of ≥ 60 kg/m^2^ in the USA. Lancet Diabetes Endocrinol 2025;13:463–465 40288378 10.1016/S2213-8587(25)00069-5

[2] Campos GM, Khoraki J, Browning MG, Pessoa BM, Mazzini GS, Wolfe L. Changes in utilization of bariatric surgery in the United States from 1993 to 2016. Ann Surg 2020;271:201–209 31425292 10.1097/SLA.0000000000003554

[3] Peterli R, Wölnerhanssen BK, Peters T, Vetter D, Kröll D, Borbély Y et al Effect of laparoscopic sleeve gastrectomy *vs* laparoscopic Roux-en-Y gastric bypass on weight loss in patients with morbid obesity: the SM-BOSS randomized clinical trial. JAMA 2018;319:255–265 29340679 10.1001/jama.2017.20897 PMC5833546

[4] Salminen P, Helmiö M, Ovaska J, Juuti A, Leivonen M, Peromaa-Haavisto P et al Effect of laparoscopic sleeve gastrectomy *vs* laparoscopic Roux-en-Y gastric bypass on weight loss at 5 years among patients with morbid obesity: the SLEEVEPASS randomized clinical trial. JAMA 2018;319:241–254 29340676 10.1001/jama.2017.20313 PMC5833550

[5] Salminen P, Grönroos S, Helmiö M, Hurme S, Juuti A, Juusela R et al Effect of laparoscopic sleeve gastrectomy *vs* Roux-en-Y gastric bypass on weight loss, comorbidities, and reflux at 10 years in adult patients with obesity: the SLEEVEPASS randomized clinical trial. JAMA Surg 2022;157:656–666 35731535 10.1001/jamasurg.2022.2229 PMC9218929

[6] Kraljevic M, Süsstrunk J, Wölnerhanssen BK, Peters T, Bueter M, Gero D et al Long-term outcomes of laparoscopic Roux-en-Y gastric bypass *vs* laparoscopic sleeve gastrectomy for obesity: the SM-BOSS randomized clinical trial. JAMA Surg 2025;160:369–377 39969869 10.1001/jamasurg.2024.7052 PMC11840683

[7] By-Band-Sleeve Collaborative Group . Roux-en-Y gastric bypass, adjustable gastric banding, or sleeve gastrectomy for severe obesity (By-Band-Sleeve): a multicentre, open label, three-group, randomised controlled trial. Lancet Diabetes Endocrinol 2025;13:410–426 40179925 10.1016/S2213-8587(25)00025-7 PMC7619233

[8] Biter LU, ‘t Hart JW, Noordman BJ, Smulders JF, Nienhuijs S, Dunkelgrün M et al Long-term effect of sleeve gastrectomy *vs* Roux-en-Y gastric bypass in people living with severe obesity: a phase III multicentre randomised controlled trial (SleeveBypass). Lancet Reg Health Eur 2024;38:100836 38313139 10.1016/j.lanepe.2024.100836 PMC10835458

[9] Brissman M, Beamish AJ, Olbers T, Marcus C. Prevalence of insufficient weight loss 5 years after Roux-en-Y gastric bypass: metabolic consequences and prediction estimates: a prospective registry study. BMJ Open 2021;11:e046407 10.1136/bmjopen-2020-046407 PMC7929824 33653767

[10] Sundbom M, Näslund I, Näslund E, Ottosson J. High acquisition rate and internal validity in the Scandinavian Obesity Surgery Registry. Surg Obes Relat Dis 2021;17:606–614 33243667 10.1016/j.soard.2020.10.017

[11] Ogrinc G, Davies L, Goodman D, Batalden P, Davidoff F, Stevens D. SQUIRE 2.0—standards for quality improvement reporting excellence-revised publication guidelines from a detailed consensus process. J Am Coll Surg 2016;222:317–323 26385723 10.1016/j.jamcollsurg.2015.07.456

[12] Riddle MC, Cefalu WT, Evans PH, Gerstein HC, Nauck MA, Oh WK et al Consensus report: definition and interpretation of remission in type 2 diabetes. Diabetes Care 2021;44:2438–2444 34462270 10.2337/dci21-0034 PMC8929179

[13] Boustani P, Sheidaei A, Mokhber S, Pazouki A. Assessment of weight change patterns following Roux en Y gastric bypass, one anastomosis gastric bypass and sleeve gastrectomy using change-point analysis. Sci Rep 2024;14:17416 39075167 10.1038/s41598-024-68480-x PMC11286853

[14] Brown R . Evaluating the effectiveness and long-term outcomes of Roux-en-Y gastric bypass *vs* gastric sleeve bariatric surgery in obese and diabetic patients: systematic review. J Am Coll Surg 2025;241:1148–1159 10.1097/XCS.0000000000001533 40709782

[15] Wölnerhanssen BK, Peterli R, Hurme S, Bueter M, Helmiö M, Juuti A et al Laparoscopic Roux-en-Y gastric bypass *versus* laparoscopic sleeve gastrectomy: 5-year outcomes of merged data from two randomized clinical trials (SLEEVEPASS and SM-BOSS). Br J Surg 2021;108:49–57 33640917 10.1093/bjs/znaa011

[16] Arapis K, Macrina N, Kadouch D, Ribeiro Parenti L, Marmuse JP, Hansel B. Outcomes of Roux-en-Y gastric bypass *versus* sleeve gastrectomy in super-super-obese patients (BMI ≥ 60 kg/m^2^): 6-year follow-up at a single university. Surg Obes Relat Dis 2019;15:23–33 30454974 10.1016/j.soard.2018.09.487

[17] Wang Y, Song Y, Chen J, Zhao R, Xia L, Cui Y et al Roux-en-Y gastric bypass *versus* sleeve gastrectomy for super super obese and super obese: systematic review and meta-analysis of weight results, comorbidity resolution. Obes Surg 2019;29:1954–1964 30953336 10.1007/s11695-019-03817-4

[18] Jain D, Sill A, Averbach A. Do patients with higher baseline BMI have improved weight loss with Roux-en-Y gastric bypass *versus* sleeve gastrectomy? Surg Obes Relat Dis 2018;14:1304–1309 30041972 10.1016/j.soard.2018.05.014

[19] Prospective Studies Collaboration . Body-mass index and cause-specific mortality in 900 000 adults: collaborative analyses of 57 prospective studies. Lancet 2009;373:1083–1096 19299006 10.1016/S0140-6736(09)60318-4 PMC2662372

[20] Sjöström L, Lindroos AK, Peltonen M, Torgerson J, Bouchard C, Carlsson B et al Lifestyle, diabetes, and cardiovascular risk factors 10 years after bariatric surgery. N Engl J Med 2004;351:2683–2693 15616203 10.1056/NEJMoa035622

[21] Mingrone G, Panunzi S, De Gaetano A, Guidone C, Iaconelli A, Leccesi L et al Bariatric surgery *versus* conventional medical therapy for type 2 diabetes. N Engl J Med 2012;366:1577–1585 22449317 10.1056/NEJMoa1200111

[22] Aminian A, Kashyap SR, Wolski KE, Brethauer SA, Kirwan JP, Nissen SE et al Patient-reported outcomes after metabolic surgery *versus* medical therapy for diabetes: insights from the STAMPEDE randomized trial. Ann Surg 2021;274:524–532 34132694 10.1097/SLA.0000000000005003 PMC8373787

[23] Haddad A, Suter M, Greve JW, Shikora S, Prager G, Dayyeh BA et al Therapeutic options for recurrence of weight and obesity related complications after metabolic and bariatric surgery: an IFSO position statement. Obes Surg 2024;34:3944–3962 39400870 10.1007/s11695-024-07489-7

[24] Cohen RV, Park JY, Prager G, Bueter M, le Roux CW, Parmar C et al Role of obesity-management medications before and after metabolic bariatric surgery: a systematic review. Br J Surg 2024;111:znae284 39612581 10.1093/bjs/znae284

[25] Mok J, Adeleke MO, Brown A, Magee CG, Firman C, Makahamadze C et al Safety and efficacy of liraglutide, 3.0 mg, once daily *vs* placebo in patients with poor weight loss following metabolic surgery: the BARI-OPTIMISE randomized clinical trial. JAMA Surg 2023;158:1003–1011 37494014 10.1001/jamasurg.2023.2930 PMC10372755

[26] Parmar CD, Bryant C, Luque-de-Leon E, Peraglie C, Prasad A, Rheinwalt K et al One anastomosis gastric bypass in morbidly obese patients with BMI ≥ 50 kg/m^2^: a systematic review comparing it with Roux-en-Y gastric bypass and sleeve gastrectomy. Obes Surg 2019;29:3039–3046 31250385 10.1007/s11695-019-04034-9

[27] Thomopoulos T, Mantziari S, Joliat GR. Long-term results of Roux-en-Y gastric bypass (RYGB) *versus* single anastomosis duodeno-ileal bypass (SADI) as revisional procedures after failed sleeve gastrectomy: a systematic literature review and pooled analysis. Langenbecks Arch Surg 2024;409:354 39579238 10.1007/s00423-024-03557-9 PMC11585492

[28] Fischer L, Nickel F, Sander J, Ernst A, Bruckner T, Herbig B et al Patient expectations of bariatric surgery are gender specific—a prospective, multicenter cohort study. Surg Obes Relat Dis 2014;10:516–523 24951069 10.1016/j.soard.2014.02.040

[29] Stenberg E, Szabo E, Ågren G, Ottosson J, Marsk R, Lönroth H et al Closure of mesenteric defects in laparoscopic gastric bypass: a multicentre, randomised, parallel, open-label trial. Lancet 2016;387:1397–1404 26895675 10.1016/S0140-6736(15)01126-5

[30] Hajibandeh S, Hajibandeh S, Abdelkarim M, Shehadeh A, Mohsin MM, Khan KA et al Closure *versus* non-closure of mesenteric defects in laparoscopic Roux-en-Y gastric bypass: a systematic review and meta-analysis. Surg Endosc 2020;34:3306–3320 32270276 10.1007/s00464-020-07544-1

