# Supplementary material for: Sleeve gastrectomy versus Roux-en-Y-gastric bypass in patients with body mass index over 50 kg/m2: international multicentre cohort
Source: BJS Open. 2026 Apr 29;10(2):zrag028. doi: 10.1093/bjsopen/zrag028 (PMC13126665; doi:10.1093/bjsopen/zrag028)
Supplement: zrag028_Supplementary_Data [file zrag028_supplementary_data.docx]

**Sleeve gastrectomy versus Roux-en-Y-gastric bypass in patients with BMI over 50kg/m²: an international multi-centre cohort**

Lars Kollmann^1^, Ilan Rosenblum^2^, Adisa Poljo^3,4^, [Pascal Probst](https://pubmed.ncbi.nlm.nih.gov/?term=Probst+P&cauthor_id=39117560) [^5^](https://pubmed.ncbi.nlm.nih.gov/39117560/#full-view-affiliation-7), [Markus K Muller](https://pubmed.ncbi.nlm.nih.gov/?term=M%C3%BCller+MK&cauthor_id=39117560)^5^, Piotr Kalinowski^6^, Muhammed Said Dalkılıç^7^, Abdullah Şişik^8^, Johanna Betzler^9^, Mirko Otto^9^, Stephanie Taha-Mehlitz^3^, Beat P. Müller^3^, Daniel M. Frey^10^, Bassey Enodien^11^, Emanuel Burri^12^, Reinhard Stoll^2^, Otto Kollmar^2^, Robert Rosenberg^2^, Florian Ponholzer^13^, Johan Friso Lock^1^, Sven Flemming^1^, Erik Stenberg^14^, Johan Ottosson^14^, Ellen Andersson^15^, Torsten Olbers^15^, Ralph Peterli^16^, Florian Seyfried^1,*^, Anas Taha^2, 17,18,*^

Collaborating: Mateusz Bartkowiak^6^, Marta Przybysz^6^ and Michał Grąt^6^, Jasmin Zeindler^2^, Marionna Cathomas^2^, Christina Bogensperger^13^, Marie-Christin Neuschmid^12^,

1. Department of General-, Visceral-, Transplantation-, Vascular-, and Pediatric Surgery; University Hospital Wuerzburg, Wuerzburg, Germany.
2. Department of Visceral Surgery, Cantonal Hospital Baselland, Liestal. Switzerland. [Ilan.Rosenblum@ksbl.ch](mailto:Ilan.Rosenblum@ksbl.ch), [Otto.Kollmar@ksbl.ch](mailto:Otto.Kollmar@ksbl.ch), [Reinhard.stoll@ksbl.ch](mailto:Reinhard.stoll@ksbl.ch) , [robert.rosenberg@ksbl.ch](mailto:robert.rosenberg@ksbl.ch)
3. Department of Visceral Surgery, Clarunis, University Center for Gastrointestinal and Liver Diseases, St. Clara Hospital and University Hospital, Basel, Switzerland. Adisa.poljo@clarunis.ch. [Stephanie.taha@clarunis.ch](mailto:Stephanie.taha@clarunis.ch)., [Beat.Mueller@clarunis.ch](mailto:Beat.Mueller@clarunis.ch)
4. Johannes Kepler University Linz, Medical Faculty, Altenberger Strasse 69, 4040 Linz, Austria
5. Department of Surgery, Cantonal Hospital Thurgau, Frauenfeld, Switzerland. [pascal.probst@stgag.ch](mailto:pascal.probst@stgag.ch).
6. Department of General, Transplant and Liver Surgery, Medical University of Warsaw, Warszawa, Poland. [piotr.kalinowski@wum.edu.pl](mailto:piotr.kalinowski@wum.edu.pl).
7. Department of General Surgery, School of Medicine, Marmara University, Istanbul, Turkey. [saiddalkilic@hotmail.com](mailto:saiddalkilic@hotmail.com).
8. Health Sciences Faculty, Gedik University, İstanbul, Turkey. [abdullahsisik@gmail.com](mailto:abdullahsisik@gmail.com)
9. Department of Surgery, Universitätsmedizin Mannheim, Medical Faculty Mannheim, Heidelberg University, Mannheim, Germany. [Johanna.Betzler@umm.de](mailto:Johanna.Betzler@umm.de), [Otto.mirko@umm.de](mailto:Otto.mirko@umm.de).
10. Department of Visceral Surgery, Cantonal Hospital Baden, Baden, Switzerland. [Daniel.Frey@ksb.ch](mailto:Daniel.Frey@ksb.ch)
11. Department of Visceral Surgery, Cantonal Hospital Glarus, Glarus; Switzerland.[bassey.enodien@ksgl.ch](mailto:bassey.enodien@ksgl.ch)
12. Department of Gastroenterology and Hepatology, Cantonal Hospital Baselland, Liestal, Switzerland Emanuel.burri@ksbl.ch
13. Department of Visceral, Transplant and Thoracic Surgery, Center of Operative Medicine, Medical University of Innsbruck, Innsbruck, Austria [florian.ponholzer@tirol-kliniken.at](mailto:florian.ponholzer@tirol-kliniken.at)
14. Department of Surgery, Faculty of Medicine and Health, Örebro University, Örebro; Sweden. [erik.stenberg@regionorebrolan.se](mailto:erik.stenberg@regionorebrolan.se)
15. Department of Surgery and Department of Clinical and Experimental Medicine, Linköping University, Norrköping, Sweden. [torsten.olbers@liu.se](mailto:torsten.olbers@liu.se)
16. Department of Clinical Research, University Hospital Basel, Switzerland, [Ralph.peterli@unibas.ch](mailto:Ralph.peterli@unibas.ch)
17. Department of Surgery, Department of Surgery, Brody School of Medicine, East Carolina University, Greenville, NC, USA.
18. Department of Biomedical Engineering, University of Basel, Basel; Switzerland.

^*^Florian Seyfried and Anas Taha contributed equally

**Funding:** None

**Corresponding author:**

Anas Taha, MD

Department of Biomedical Engineering, Faculty of Medicine; University of Basel, Hegenheimermattweg 167C, 4123 Allschwil, Switzerland,

E-Mail: Anas.taha@unibas.ch

Phone: +41 61 207 5402

**Supplementary Materials – Index**

| **Supplementary Methods** |  |  |
| --- | --- | --- |
| Statistical analyses | *page 4/5* |  |
|  |  |  |
| **Supplementary Results** |  |  |
| Baseline cohort | *page 5* |  |
| Completed follow up cohort | *page 5* |  |
|  |  |  |
|  |  |  |
|  |  |  |
| **Supplementary Figures and Tables** |  |  |
| Supplementary Table 1: Distribution of participating centers. | *page 5/6* |  |
| Supplementary Table 2: Baseline characteristics before propensity matching. | | *page 6* |
| Supplementary Table 3: Results at 12 months cohort before propensity matching. | | *page 6* |
| Supplementary Table 4: Results at 5 years cohort before propensity matching.  . | | *page 6/7* |
| Supplementary Table 5: Baseline characteristics subgroup 5y FU completed.  . | | *page 7* |
| Supplementary Table 6: Results at 12 months subgroup 5y FU completed.  . | *page 7* |  |
|  |  |  |

**Supplementary Methods**

All statistical analyses were performed using SPSS Statistics 29 (IBM, Armonk, NY). All statistical analyses were performed using SPSS Statistics 29 (IBM, Armonk, NY). Descriptive data are reported as means with standard deviations, unless otherwise stated. The propensity variable for 1:1 matching with the nearest neighbour was calculated by logistic regression analysis incorporating the selected covariates. A caliper of <0.05 of the standard deviation (SD) of the logit of the propensity was accepted. Covariates for propensity matching were age, sex, T2D and BMI as they were the most important factors regarding the outcome variables and were the most complete available for the majority of the cases. Comparisons between the analysed cohorts were performed using chi-square test, Fisher’s exact test, Mann–Whitney U-test or a one-way analysis of variance in accordance with data scale and distribution. The level of statistical significance was 0.05 (two-sided). All statistical analyses were performed for the baseline cohort (including patients with missing follow-up) and for the subgroup of patients only with completed five years follow up to control for possible selection bias. These are shown in the supplementary tables.

**Supplementary Results**

The baseline characteristics and outcomes in the overall cohort of 8,160 patients showed trends in %TBWL, BMI, and the rate of patients with suboptimal weight loss similar to those observed in the propensity matched cohort. To further assess possible bias we decided to calculate all parameters for the baseline cohort before matching. The results were overall very similar to the matched cohort. These data are presented in Supplementary Tables 2, 3 and 4.

To evaluate the effect of a possible bias by lost to follow up patients we then did the analyses for the subgroup of patients only with completed five- year follow up. The results also did not differ from the overall or matched cohort. Results are shown in Supplementary Tables 5 and 6.

**Supplementary Figures and Tables**

Supplementary Table 1: Distribution of participating centers.

| **Center** | Total  n =3976  (%) | Sleeve  n=1988  (%) | RYGB  n=1988  (%) |
| --- | --- | --- | --- |
| Claraspital Basel (Basel, Switzerland) | 246 (6.2) | 168 (8.5) | 78 (3.9) |
| Kantonsspital Frauenfeld (Frauenfeld, Switzerland) | 44 (1.1) | 14 (0.7) | 30 (1.5) |
| Universitätsspital Innsbruck (Innsbruck, Austria) | 39 (1.0) | 19 (1.0) | 20 (1.0) |
| Kantonsspital Baselland (Liestal, Switzerland) | 22 (0.6) | 18 (0.9) | 4 (0.2) |
| NOVA University Lissabon (Lissabon, Portugal) | 3 (0.1) | 0 (0.0) | 3 (0.2) |
| Universitätsmedizin Mannheim (Mannheim, Germany) | 334 (8.4) | 307 (15.4) | 27 (1.4) |
| Marmara University Istanbul (Istanbul, Turkey) | 113 (2.8) | 112 (5.6) | 1 (0.1) |
| University Hospital Würzburg (Würzburg, Germany) | 277 (7.0) | 198 (10.0) | 79 (4.0) |
| Swedish National Registry* | 2800 (70.4) | 1074 (54.0) | 1727 (86.8) |
| University Warschaw (Warschaw, Poland) | 19 (0.5) | 19 (1.0) | 0 (0.0) |
| Kantonsspital Wetzikon (Wetzikon, Switzerland) | 79 (2.0) | 59 (3.0) | 20 (1.0) |

*: participating centers: Örebro University, Linköping University

Supplementary Table 2: Baseline characteristics before propensity matching.

| **Patient Characteristics** | Total  (n =8160) | Sleeve  (n=2061) | RYGB  (n=6099) | p-Value |
| --- | --- | --- | --- | --- |
| Age median; Range | 39 (14-76) | 40 (16-76) | 38.5 (14-76) | <.001 |
| Sex m/f (%) | 2721:5439 (33.3:66.7) | 720:1341 (34.9:65.1) | 2001:4098 (32.8:67.2) | .041 |
| BMI at baseline (kg/m²) median (range) | 54.7 (50-100) | 56.2 (50-100) | 54.2 (50-83.9) | <.001 |
| Diabetes at baseline (%)   - Insuline dependent | 1518 (18.9)  85 (5.3) | 527 (26.4)  49 (5.4) | 991 (16.4)  36 (5.2.) | <.001  .480 |

* RYGB: Roux-en-y-gastric bypass; m/f: male/female; kg: kilogram;

Supplementary Table 3: Results at 12 months cohort before propensity matching.

| **Postoperative Results** | Total  (n =5512) | Sleeve  (n=930) | RYGB  (n=4582) | p-Value |
| --- | --- | --- | --- | --- |
| BMI at 12 months; median (range) | 37.7 (20-64.7) | 39.2 (22-64.7) | 37.2 (20-60.8) | <.001 |
| Diabetes at 12 months (351 cases) | 319 (5.8) | 82 (8.8) | 237 (5.2) | <.001 |
| %TBWL at 12 months; median (range) | 31.3 (2.2-63.7) | 30.3 (2.2-63.7) | 31.6 (6.5-60.3) | <.001 |
| BMI >40kg/m² at 12 months | 1609 (28.4) | 622 (42.6) | 987 (23.5) | <.001 |
| BMI >50kg/m² at 12 months | 130 (2.3) | 83 (5.7) | 47 (1.1) | <.001 |

* BMI: body mass index; RYGB: Roux-en-y-gastric bypass; m/f: male/female; kg: kilogram; % TBWL: percent total body weight loss.

Supplementary Table 4: Results at 5 years cohort before propensity matching.

| **Postoperative Results** | Total  (n =2214) | Sleeve  (n=319) | RYGB  (n=1895) | p-Value |
| --- | --- | --- | --- | --- |
| BMI at 5 years | 39.3 (20-67.2) | 41.7 (22-67.2) | 38.7 (20-63.8) | <.001 |
| Diabetes at 5 years | 168 (7.6) | 41 (12.9) | 127 (6.7) | <.001 |
| %TBWL at 5 years; mean (range) | 27.7 (-6.6-62.9) | 25.5 (-4.8 -56) | 28.3 (-6.6-62.9) | **<.001** |
| BMI>40kg/m² at 5 years | 851 (41.4) | 247 (57.4) | 604 (37.2) | <.001 |
| BMI>50kg/m² at 5 years | 114 (5.6) | 59 (13.7) | 55 (3.4) | <.001 |
| Follow up rate at 5 years (%) | 27.1 | 15.4 | 31.1 | <.001 |

* BMI: body mass index; RYGB: Roux-en-y-gastric bypass; m/f: male/female; kg: kilogram; % TBWL: percent total body weight loss.

Supplementary Table 5: Baseline characteristics subgroup 5y FU completed.

| **Patient Characteristics** | Total  (n =2054) | Sleeve  (n=430) | RYGB  (n=1624) | p-Value |
| --- | --- | --- | --- | --- |
| Age; median (range) | 40.6 (17-76) | 41.4 (17-76) | 40.5 (18-71) | .147 |
| Sex m/f (%) | 668:1386 (32.5:67.5) | 138:292 (32.1:67.9) | 530:1094 (32.6:67.4) | .440 |
| BMI at baseline (kg/m²) median (range) | 54.5 (50-79) | 56.1 (50-79) | 54.0 (50-76.2) | <.001 |
| Diabetes at baseline   - Insuline dependent | 379 (18.6)  29 (7.0) | 108 (25.6)  15 (7.2) | 271 (16.8)  14 (.6.7) | <.001  .495 |

* BMI: body mass index; RYGB: Roux-en-y-gastric bypass; m/f: male/female; kg: kilogram;

Supplementary Table 6: Results at 12 months subgroup 5y FU completed

| **Postoperative Results** | Total  (n=1765) | Sleeve  (n=385) | RYGB  (n=1380) | p-Value |
| --- | --- | --- | --- | --- |
| BMI at 12 months | 37.9 (21-62.6) | 39.0 (22-62.5) | 37.6 (21–59.7) | <.001 |
| Diabetes at 12 months (140 cases) | 27 (19.3) | 13 (26.5) | 14 (15.4) | .087 |
| %TBWL at 12 months; mean (range) | 30.5 (5.9-62.3) | 30.5 (5.9-62.3) | 30.5 (6.5-60.1) | .884 |
| BMI >40kg/² at 12 months | 524 (29.7) | 162 (42.1) | 362 (26.2)) | .006 |
| BMI >50kg/² at 12 months | 36 (2.0) | 24 (6.2) | 12 (0.9) | .001 |

* BMI: body mass index; RYGB: Roux-en-y-gastric bypass; kg: kilogram; % TBWL: percent total body weight loss.
